# microRNA-Mediated Encoding and Decoding of Time-Dependent Signals in Tumorigenesis

**DOI:** 10.3390/biom12020213

**Published:** 2022-01-26

**Authors:** Simone Tealdi, Elsi Ferro, Carlo Cosimo Campa, Carla Bosia

**Affiliations:** 1 Italian Institute for Genomic Medicine, c/o IRCCS, Str. Prov. le 142, km 3.95, 10060 Candiolo, Italy; simone.tealdi@polito.it (S.T.); elsi.ferro@polito.it (E.F.); carlocosimo.campa@iigm.it (C.C.C.); 2 Department of Mechanical and Aerospace Engineering, Politecnico di Torino, Corso Duca degli Abruzzi 24, 10129 Torino, Italy; 3 Department of Applied Science and Technology, Politecnico di Torino, Corso Duca degli Abruzzi 24, 10129 Torino, Italy; 4 Candiolo Cancer Institute, FPO-IRCCS, Str. Prov. le 142, km 3.95, 10060 Candiolo, Italy

**Keywords:** microRNA-mediated motifs, temporal dynamics, tumorigenesis, feed-forward loop, feedback loop, p53, Myc

## Abstract

microRNAs, pivotal post-transcriptional regulators of gene expression, in the past decades have caught the attention of researchers for their involvement in different biological processes, ranging from cell development to cancer. Although lots of effort has been devoted to elucidate the topological features and the equilibrium properties of microRNA-mediated motifs, little is known about how the information encoded in frequency, amplitude, duration, and other features of their regulatory signals can affect the resulting gene expression patterns. Here, we review the current knowledge about microRNA-mediated gene regulatory networks characterized by time-dependent input signals, such as pulses, transient inputs, and oscillations. First, we identify the general characteristic of the main motifs underlying temporal patterns. Then, we analyze their impact on two commonly studied oncogenic networks, showing how their dysfunction can lead to tumorigenesis.

## 1. Introduction

Cell fate changes are the driving force of cancer [1], and they are induced by changes in gene expression patterns. These patterns are the collective outcome of interactions between individual genes, whose activity is determined by the topology and dynamics of gene regulatory networks. Turning genes on or off and changing their levels above or below a threshold can dynamically lead the gene expression network toward new “equilibrium states”, thereby determining cell fate changes. This idea is well captured in Waddington’s notion of “epigenetic landscape”, a concept first introduced in the 1950s as a metaphor to explain the genetically mediated modulation of development [2,3,4].

**Figure 1:**
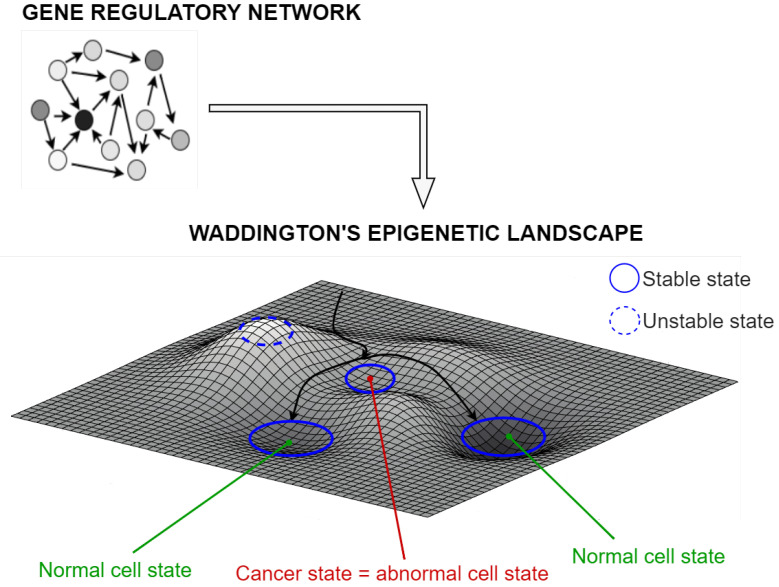
Representation of Waddington’s epigenetic landscape. The landscape is depicted with random morphology aimed at merely exemplifying purposes. Each point in the epigenetic landscape represents a state of the genetic network, defined by the expression levels of each one of the genes constituting the network. Changes in gene expression can lead to shifts in the epigenetic landscape. As the network’s collective expression pattern changes, the point defining its state shifts through the landscape until settling down into a new equilibrium state. Examples of the network state’s time evolution are depicted with black arrows. Stable equilibrium states, corresponding to valleys, are highlighted with blue solid circles, whereas unstable equilibrium states, associated to hills, are highlighted with blue dashed circles. Both normal cell states (highlighted with green circles) and abnormal cancer cell states (indicated with red circles) can be found among stable equilibrium states.

According to Waddington, each configuration of the interconnected gene activities corresponds to a “gene expression state” of the network, that is, a state defined by the expression levels of each one of the genes constituting the network. Any gene expression pattern of the network, as a whole, maps to a single point in the space of all possible network states. A perturbation of gene expression can alter the state of the network, leading to a shift in Waddington’s landscape along a trajectory dictated by the connectivity and magnitude of regulatory interactions. Thereafter, the network state evolves in time until it settles down into a new equilibrium state, that is, a new stable pattern of gene expression. A gene expression pattern is defined as stable if gene expression levels are kept stationary when subject to fluctuations, meaning that the system is unlikely to move toward a new state. Mathematically, the state space can be viewed as a multi-dimensional landscape where equilibrium states correspond to pits, also called “potential wells” in the state space formalism. In such a landscape, a valley represents a stable gene expression state, whereas hills correspond to unstable states, namely expression patterns more sensitive to fluctuations, where the system is more likely to move toward a new gene expression state (see Figure 1). However, transitions between cell states are triggered by signals that cause changes in the network’s gene expression levels [5,6,7], moving the system toward a new valley of Waddington’s landscape.

Abnormal cell states, such as cancer [8], belong to the epigenetic landscape as any other possible stable state of the network (Figure 1). Transitions from a normal to an abnormal state are facilitated by genetic mutations, which can change the expression pattern of a set of genes [9], or by non-genetic events [10], such as regulatory signals, which can generate fluctuations in gene activity [11]. These perturbations often confer to cells’ pro-tumorigenic features such as unregulated proliferation, avoidance of apoptotic death, abnormal migration, and loss of differentiation.

A growing number of studies have unveiled that the temporal dynamics of regulatory signals encodes the key information that determines a network’s outcoming gene expression pattern [12]. With temporal dynamics is intended the shape by which the concentration of signaling molecules changes over time. In this sense, the information is encoded in the frequency, amplitude, duration, and other features of the signal. Thus, transitions between cell states appear to be controlled by the decoding of complex temporal variables rather than by simple instantaneous signal levels. This assessment adds a new layer of complexity to gene regulatory networks and implies that characterizing the actors of a network (in terms of genes and interactions amongst genes) as well as their temporal dynamics can lead to a better understanding of how transitions to abnormal cancerous cell states occur.

microRNAs (miRNAs), small non-coding RNAs with a length of ∼22 nucleotides, have caught the spotlight among the diverse members of regulatory networks that play a role in tumorigenesis [13]. MiRNAs mainly function by repressing gene expression at the post-transcriptional level [14]. Specifically, they bind to their target gene’s messenger RNA by Watson–Crick base pairing with complementary sequences called MiRNA response elements (MREs), which are located either at 3′ or 5′ untranslated regions of the mRNA. The degree of MRE complementarity determines whether mRNA translation is inhibited (poor complementarity) or transcript degradation is enhanced (high complementarity) [15], thereby leading to different degrees of repression. The progressive uncovering of miRNA-mediated regulatory connections revealed that single miRNAs are able to regulate multiple target genes and single genes can be subject to the simultaneous regulation of several miRNAs [16,17,18]. Furthermore, miRNAs can induce titration in target gene expression [19] and generate competition for binding among different RNA species [20,21].

MiRNAs play crucial roles in many biological processes related to cancer, such as cell fate determination [22], proliferation [23], and cell death [24]. Indeed, their dysregulation has been linked to several cancer types [25], with notable connections to cancer progression [26] and metastasis [27], where they can exert either tumor-suppressive or oncogenic functions. In the past years, studies concerning miRNA-mediated regulatory networks have provided a deeper understanding of the interplay between post-transcriptional and transcriptional layers of regulation [28]. This helped to address how the dysregulation of miRNA function can favor the transition of cells to abnormal “cancer” states [29] by topographically altering the epigenetic landscape or by causing the network’s expression state to change its position within the landscape.

Many works reviewed the topological features and the equilibrium properties of miRNA-mediated motifs (i.e., miRNA-mediated regulatory patterns that are over-represented in regulatory networks) [30,31]. However, to our knowledge, little effort has been devoted to reviewing their temporal dynamic properties and how these can affect the resulting gene expression patterns.

Here, we review the research efforts on the role of miRNAs within gene regulatory motifs, emphasizing their role in tumorigenesis. Specifically, we focus on motifs that exploit temporal signal features. Special attention is devoted to pulsing signals, transient inputs, and oscillations. First, the general features of these motifs are outlined. In particular, how these features contribute in converting information into time-dependent signals (encoding) and in translating it into distinct phenotypic responses (decoding) is elicited. Next, the p53- and the Myc-controlled genetic networks are brought as exemplary cases of oncogenic pathways where miRNAs mediate the encoding and decoding of time-dependent signals. Moreover, the tumorigenic role of miRNAs within these networks is highlighted.

## 2. Feedforward Loops and Feedback Loops Interpret Temporally Encoded Patterns

The response of a gene regulatory motif depends strongly on the properties of input signals. These properties have been proven to be crucial in determining the behaviors of single cells, as well as at the population level [32,33,34]. Feedforward loop (FFL) and feedback loop (FBL) are the recurrent motifs by which genetic networks can be decomposed when the properties of input signals are attributable to temporal dynamics [35,36].

### 2.1. Feedforward Loops Differentiate between Pulsatile and Transient Signals

**Figure 2:**
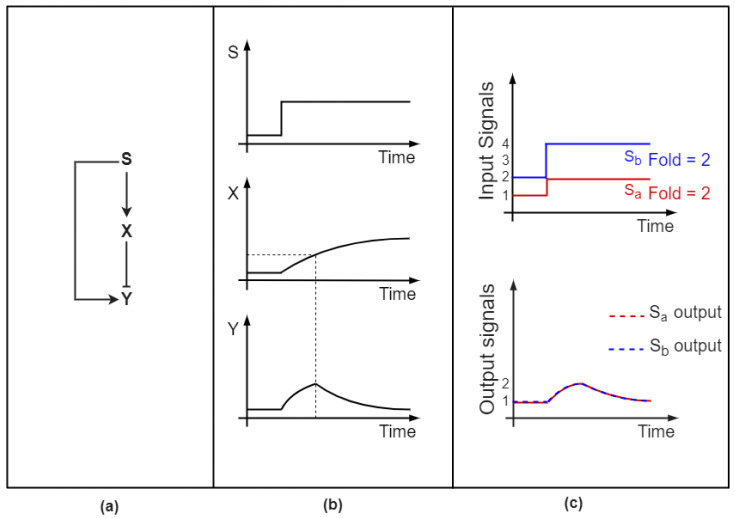
Representation of the incoherent type-1 feedforward loop (I1-FFL) and its signal-decoding properties. Edges with a pointed head represent positive (activatory) regulation, whereas edges with a flat head indicate negative (repressive) regulation. (**a**) I1-FFL: a master regulator S activates both a target output gene Y and a repressor X, which in turn targets Y. (**b**) Example of pulse-like response generation: a transient stimulus S induces the production of both X and Y. However, the output’s repression threshold is typically higher that its activation threshold. Thus, a Y increase is allowed until X reaches its threshold concentration (dashed line), leading the output’s level to drop. (**c**) Example of fold change detection: two input signals characterized by equal fold-change but different absolute levels lead to identical output responses.

FFLs are regulatory motifs that involve three genes: a master regulator which targets a gene both directly and indirectly through the regulation of a third player that targets the same gene [37]. Each of the regulatory paths composing this motif can be represented by negative (inhibitory) or positive (activatory) regulation, meaning that there exist eight possible combinations. FFLs are usually classified into two groups depending on the sign of the direct and indirect path of regulation: if both have the same sign, the FFL is said to be coherent, if they have opposite signs, the FFL is said to be incoherent [37]. FFLs, originally found in *E. coli* [38,39], have been observed in many different organisms including yeast [40] and mammals [39,41]. Since our aim is to analyze motifs that significantly exploit temporal dynamics, we will focus on the single type of FFL able to distinguish oscillatory from transient input signals, that is the so-called Incoherent type-1 FFL (I1-FFL) [42,43] (Figure 2a).

In this regulatory circuit, the upstream input directly activates the output gene and indirectly inhibits the same gene by triggering the expression of a repressor [37]. An environmental signal can lead to the expression of the master regulator, which in turn leads to the production of both the target gene and the repressor, which accumulate over time. However, due to the target promoter’s different activation and repression thresholds, the output molecule is initially produced without interference by the repressor. Once the repressor reaches the threshold concentration necessary to effectively exert its inhibitory action, the target’s production decreases, leading its amount to drop. This results in a pulse-like temporal dynamics (Figure 2b), as demonstrated by the perturbation of synthetic circuits in *E. coli* [44].

The ability to generate a transient response to an input perturbation has a meaningful adaptive function [45,46]. Indeed, the I1-FFL allows the output gene to react transiently to fluctuations to then return to its pre-stimulated output levels, even if the stimulus persists [47]. This property has been also observed specifically within miRNA-mediated I1-FFLs: Strovas and Colleagues demonstrated that a minimal I1-FFL where a gene encodes for its own repressor miRNA, i.e., an intronic miRNA-mediated loop, exhibits adaptation. This simple motif was shown to efficiently buffer protein production against fluctuations in transcription, returning to pre-stimulus levels after a transient pulse [48]. Hence, temporal adaptation provides the system with the ability to maintain homeostasis in the presence of environmental perturbations [49]. Given its functional advantage, the I1-FFL is one of the most prevalent endogenous motifs [39]. Indeed, pulse-like gene expression has been observed in a wide range of organisms, from bacteria to mammals [50,51,52,53], in response to perturbations of the surrounding environment [54].

Besides implementing adaptation, the I1-FFL is able to detect signal fold-changes according to Weber’s law: its response relies on the relative rather than absolute variation of the input [55]. Indeed, the amplitude and duration of its output gene expression dynamics depend only on the input’s fold-change, disregarding absolute signal levels. Figure 2c illustrates this behavior: two signals with identical temporal fold-change but different absolute levels lead to the same output. Fold-change detection is widely exploited by mammalian cells [56,57] to recognize and respond to input signals that rise sufficiently above noise with respect to a background signal level. This allows one to avoid reacting to false-positive stimuli [55]. Both adaptation and fold-change detection properties have also been predicted by mathematical modelling of a miRNA-mediated I1-FFL [58]. Interestingly, the minimal I1-FFL mediated by an intronic miRNA showed the highest efficiency in adapting to basal stimulus levels and detecting relative input changes. Once more, the I1-FFL displays a stochastic and environmental fluctuation-buffering feature.

The discussed properties confer to this motif the interesting ability to differentiate between oscillatory and transient input signals. Transient signals lead to a single gene expression pulse due to adaptation: the output product’s amount settles back to its basal level, disregarding the persistence of the stimulus. Conversely, as investigated both analytically and numerically by Cerone and Neufeld, a sustained pulsatile input generates downstream oscillatory output [59]. The authors analyzed the transcriptional activity of genes regulated by I1-FFL in response to oscillations in the concentration of input transcription factors (TFs). In the presence of an oscillating master TF, the I1-FFL acts as an oscillation detector by exploiting the gap between the output gene’s activation and repression thresholds. Whenever the concentration of the TF overcomes the lower threshold concentration, necessary for effective activation, the output expression increases. When a further rise of the TF allows one to reach the repression threshold concentration needed to initiate the action of the repressor, the target gene expression decays. Thus, the recurring signal rises and drops carried along by an oscillatory input lead to repetitive increases and decays of the output. Interestingly, the slower timescales characterizing miRNA kinetics with respect to transcriptional regulation introduce a time delay between the output’s activation by the master TF and the repressive miRNA action. This gap can create a temporal avoidance effect between the expression of the repressor and that of the output, thereby giving rise to opposing time-dependent oscillations [60].

In general, oscillatory input dynamics can maximize the protein production within I1-FFLs: the amount of proteins achieves a maximum according to the shape and the features of external periodic signals [61], highlighting once more the key role of temporal dynamics. Indeed, numerical simulations revealed that I1-FFLs, stimulated with a train of square pulses, maximize the output protein expression over one period, provided that the pulse and the interpulse time intervals are adequately chosen.

MiRNAs can also act as temporal oscillation-buffers within I1-FFLs [62]. Kim and colleagues [63] showed experimentally that miRNA *lin-4* can dampen the periodic oscillations of its target *lin-14* by oscillating in synchronous pulses. These findings suggested that both *lin-14* and *lin-4* expression was driven by an upstream oscillating TF, thus describing a I1-FFL. Moreover, this dampening was demonstrated as crucial for the normal progression of cell fate decision processes that underlie *C. Elegans* development.

### 2.2. Feedback Loops: From Memory Effects to Oscillating Behaviors

**Figure 3:**
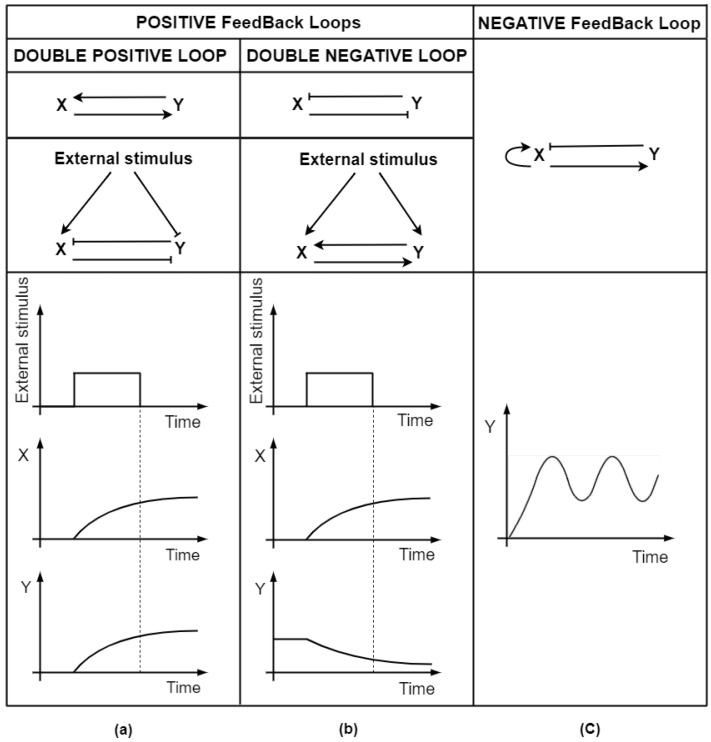
Representation of positive and negative feedback loops (P-FBL and N-FBL, respectively) and their signal-decoding properties. Edges with a pointed head represent positive (activatory) regulation, whereas edges with a flat head indicate negative (repressive) regulation. P-FBLs can be divided into two subgroups: double-positive loops and double-negative loops. (**a**) Upper panel: representation of the double-positive loop. Both regulatory paths are characterized by a positive sign. Middle panel: example of the memory-preservation property of the double-positive loop. A transient input stimulus that triggers the production of both X and Y leads their steady-state expression to remain switched on even after the deactivation (dashed line) of the input. (**b**) Upper panel: representation of the double-negative loop. Both regulatory paths are characterized by a negative sign. Middle and bottom panel: example of the memory-preservation property of the double-negative loop. A transient input stimulus that activates both X and Y leads their mutually exclusive steady-state expression, where one gene is active whereas the other is switched off, to remain locked even after the deactivation (dashed line) of the input. (**c**) Upper panel: representation of the N-FBL: regulatory paths are characterized by opposite signs. Typically, one of the two players is characterized by a positive auto-regulatory loop. Bottom panel: example of the oscillation-generating property of the N-FBL. Sustained oscillations can be generated by the N-FBL, provided that the two players are characterized by different timescales of action.

The minimal FBL is constituted by two reciprocally regulated genes. Similar to FFLs, FBLs are classified based on the sign of edges representing interactions between the two genes [64]. Positive-feedback loops (P-FBLs) and negative-feedback loops (N-FBLs) are characterized, respectively, by positive and negative overall signs of the motif. Therefore, while P-FBLs can present either double-activatory (Figure 3a, upper panel) or double-inhibitory (Figure 3b, upper panel) interactions, referred to as double-positive and double-negative loop, respectively, N-FBLs always occur with one negative and one positive interaction (Figure 3c, upper panel).

In the double-positive loop, temporal dynamics can either lead to simultaneous activity or concomitant inactivity of the two genes at equilibrium, thereby generating an “all-or-none” outcome. Conversely, two genes connected by a double-negative interaction can generate a “winner-takes-all”-like response, where one gene is active whereas the other is switched off.

Both positive FBL types display memory-preservation properties, as a transient signal can irreversibly block the system in a given steady state. This means that P-FBLs provide the memory of an input signal, even after it has been turned off. For instance, when an external upstream signal activates both genes within a double-positive loop (Figure 3a, middle panel), their steady-state output protein production remains locked at a certain level even after the deactivation of the stimulus [64] (Figure 3a, bottom panel).

A similar gene expression-locking behavior is exhibited by the double-negative FBL, which functions as a toggle-switch between two different fates. Indeed, if stimulated by an upstream regulatory signal (Figure 3b, middle panel), its winner-takes-all output persists in the absence of the original signal [65] (Figure 3b, bottom panel). Genes involved in P-FBLs can further strengthen memory preservation by employing regulatory self-loops [64]. Such memory-conservation ability makes P-FBLs suitable for transducing signals into cell fate decisions [66,67], driving typically irreversible processes such as those involved in development [68,69].

MiRNAs appear to be heavily employed as endogenous regulators in this latter motif: typically, a miRNA that post-transcriptionally represses a TF is also transcriptionally inhibited by the same TF. Osella and collaborators conducted an extensive theoretical study on miRNA-mediated toggle-switches, highlighting their potential contribution to cell differentiation [70]. It was demonstrated that not only the equilibrium properties of this switch are recovered in the specific case of miRNA mediation but also that miRNA action improves this circuit’s stability: the typical switch-like outcome where the two gene activities are mutually exclusive shows increased robustness against noise, thus exemplifying the functional advantage of miRNA-mediated toggle-switches in cell fate decision.

On the other hand, N-FBLs are associated mostly with the maintenance of homeostasis, as their wiring pattern is suited to stabilize gene expression and to minimize fluctuations coming from sudden perturbations [71]. Indeed, an external signal that perturbs either one of the two genes typically leads to their anticorrelated oscillatory dynamics, which is rapidly dampened in time and leads both the activator and the repressor to stable equilibrium levels of expression [64]. Typical dynamic features of the N-FBL have been observed not only at the level of transcriptional interactions but also in other kinds of molecular interplays [72], including miRNA-mediated post-transcriptional regulation. Cancer-related examples include the N-FBL between miR-200c and the protein product of TP53, affecting tumor progression in prostate cancer [73], and the one involving the TGF-β1 growth factor and miR-145, whose normal function dampens the acquisition of fibroblast phenotype [74].

The separation of timescales implied by the interconnection of transcriptional and post-transcriptional levels of interaction [75] can enhance the ability to maintain homeostasis, as this delay stabilizes the temporal dynamics of the motif’s components and avoids fluctuations that promote instability. Zhong-Ru and collaborators demonstrated theoretically that regulation by miRNAs within N-FBLs can act as an expression-stabilizing element by suppressing oscillations [76].

Conversely, provided that an external oscillatory signal stimulates one of the two players or the activator enhances its own activity [77] (Figure 3c, middle panel), the separation of timescales also allows N-FBLs to generate sustained oscillations, (Figure 3c, bottom panel). For instance, time-lapse imaging and computational simulations showed that an N-FBL drives oscillations in the nuclear factor kappa light chain enhancer of activated B cells (NF-kB) translocation [78]. Similarly, the time delay is exploited by miRNA-mediated N-FBLs to generate periodic expression oscillations of circadian clock-related genes such as PER [79].

## 3. microRNA-Mediated Gene Regulatory Networks and Temporal Dynamics

In this section, miRNA-mediated gene regulatory networks with TP53’s [80] and MYC’s protein products [81] (the cellular tumor antigen p53 and the proto-oncogene Myc, respectively) as central nodes are analyzed. P53 is a tumor suppressor that plays crucial roles in the regulation of the cell cycle, apoptosis, and genomic stability [82]. Interestingly, the TP53 gene is the most frequently mutated gene in human cancer cells [83]. The oncoprotein MYC is a potent driver of many human cancers. Its function regulates promoter binding and epigenetic modifications, as well as post-transcriptional processes [84].

First, an introduction on p53 and Myc functions is given, describing the actors involved along with their biological roles. Then, how the encoding and decoding of temporally varying signals acts in p53-, Myc-, and miRNA-involving regulatory circuits, allowing transitions between cell states, is explained. Specifically, we focus on how miRNAs can affect p53 and Myc pathways and how their dysregulation can lead to tumorigenesis.

### 3.1. microRNAs and p53: Cooperation to Guard the Genome

Throughout their life cycle, cells are constantly exposed to a variety of environmental stressors that lead them to face more or less severe damage to DNA. Endogenous damages derived from metabolic byproducts such as reactive oxygen species (ROS) or exogenous damages caused by ultraviolet and ionizing irradiation can require cells to attain fate decisions to repair their genome.

MiRNAs play a significant role in allowing cells to deal with environmental stresses [85,86], with pivotal connections with the stress response pathway dominated by the nuclear TF p53. P53 is one of the main actors on which cells rely to determine their fate following DNA damage [80]. Cell cycle arrest, apoptosis, or senescence can be promoted depending on whether p53 exhibits a transient or a pulsing expression [87,88,89,90].

If DNA damage signals are absent, the cellular amount of p53 is kept low thanks to proteasomal degradation mediated by the ubiquitin protein ligase (MDM2) [91,92,93]. When DNA damage occurs, p53 starts to accumulate in the nucleus due to post-translational modifications such as phosphorylation and acetylation that prevent its degradation. Indeed, such chemical modifications give rise to the dissociation of MDM2 from p53 [87], leading the latter to assume its active form. The active form of p53 regulates the expression of a set of target genes that can cause cell cycle arrest, senescence, or cell death depending on the extent and type of DNA damage [94]. If the damage is slight, cell cycle arrest allows cells to repair DNA and re-enter the normal cell cycle when the repair is complete. Conversely, if the DNA damage is severe, p53 leads to apoptotic processes, thus eliminating cells and avoiding that the damaged DNA is transferred to daughter cells.

The ability to decide between the life and death of a cell, which is crucial for preserving the integrity of DNA, makes p53 a perfect guardian of genome. Therefore, p53 has been proposed to be one of the first defenses against cancer, as it prevents the accumulation of potentially dangerous mutations [95]. In support of this hypothesis, mutations in the gene encoding p53, TP53, are related to a wide spectrum of human cancers [96,97], thus confirming its tumor-suppressive role.

However, it is not the simple level of p53 expression that determines the final cell-fate outcome following DNA damage but rather its temporal pattern. Indeed, it was shown that in response to DNA breaks caused by γ-irradiation, the concentration of p53 exhibits a pulsing temporal dynamics with fixed amplitude and frequency, which leads to temporary cell cycle arrest [98]. Conversely, ultraviolet (UV) radiation triggers a transient p53 expression with dose-dependent amplitude and duration, which results in either senescence or cell death [99]. The hypothesis that cells choose different fates based on p53 dynamics was confirmed by altering the gene’s temporal expression and observing how downstream cell fate decisions were affected [100].

**Figure 4:**
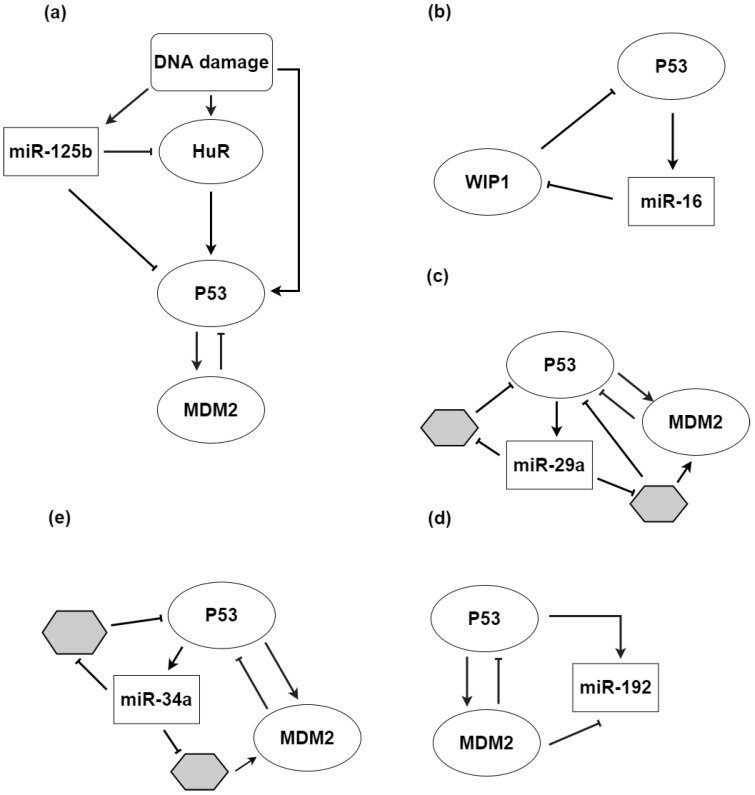
Examples of p53-governed regulatory networks involving miRNAs. Edges with a pointed head represent positive (activatory) regulation, whereas edges with a flat head represent negative (repressive) regulation. (**a**) Representation of interactions between p53, miR-125b, MDM2, and the RNA-binding protein HuR. Mir-125b contributes to the p53 pulse generation subsequent to UV irradiation by concomitantly targeting p53 and Hur. (**b**) Representation of the positive feedback loop between p53 and Wip1, mediated by miR-16. MiR-16 contributes to the discrimination between cell death and senescence. (**c**–**e**) Networks involving miR-192, miR-34a, miR-29a, and the p53-MDM2 module. Grey hexagons represent further elements constituting the networks. The three miRNAs confer robustness to the networks by tuning p53 temporal oscillations.

MiRNAs contribute to the p53 response to UV-irradiation [101]. Computational modelling and experimental validation allow one to demonstrate that miR-125b interacts with p53 and Hur (Figure 4a), a protein that increases p53 expression following DNA damage. Hur affects p53 mRNA stability and translation by binding to the transcript’s 3′UTR, and it enhances p53 protein synthesis [102]. In this context, miR-125b accomplishes the important function of stabilizing both HuR and p53 protein synthesis levels by concomitantly targeting them, thereby contributing to the generation of the UV-associated pulsing behavior of p53 [103].

The choice between oscillatory and transient p53 dynamics relies on the feedback mechanism triggered, as two distinct wiring patterns are stimulated depending on whether DNA damage is caused by γ-irradiation or UV radiation [99]. Both wiring patterns rely on PI3 kinase-related kinases (ATM or ATR) that transmit the damage signal to p53 in order to activate two negative FBLs. The first FBL, which involves p53 and the ubiquitin ligase MDM2, is affected by an additional feedback between p53 and ATM mediated by Wip1, which leads to p53 temporal oscillation [99]. The second FBL, linking p53 and the phosphatase Wip1, simply leads to a single p53 expression pulse.

As a mathematical model [104,105] demonstrated, miR-16 mediates the p53-Wip1 FBL, thereby tuning the temporal p53 pattern that leads to preference toward either apoptosis or senescence [106] (Figure 4b). Moreover, p53 promotes the transformation of primary miR-16 transcripts into precursor miR-16, thus increasing its mature levels [107]. Further FBLs including miRNA repression contributed by conferring robustness to p53 activity oscillations within a cell population, hence directly supporting cell fate decision [108]: miR-192, miR-34a, and miR-29a were shown to tune temporal expression pulses of the TF by acting within three P-FBLs (Figure 4c–e) that involve once more the p53-MDM2 module and several other proteins affected by p53 pulsing. Interestingly, miR-192 knockdown leads to the collapse of p53 pulses, whereas the silencing of miR-34a and miR-29a mildly affects p53 temporal dynamics.

Previous to the final cell fate choice, the either oscillatory or transient p53 temporal pattern is further decoded by an I1-FFL: the master TF p53 activates both the pro-apoptotic gene PUMA [109] and Slug, a TF that in turn inhibits PUMA expression [110]. In response to p53 temporal pulsing, the output pro-apoptotic gene activity has been observed to oscillate by mirroring p53 behavior [111]. Conversely, according to theoretical speculations [44] on I1-FFL properties, a transient p53 input would lead to the single pulse-like activation of PUMA, subsequently resulting in cell death. Notably, the p53-mediated apoptosis process involves both miR-34 [112], one of the first miRNAs found to be regulated by the gene, and miR-125b [113].

Further evidence indicates that miRNAs act by offering robustness to the p53 stress-response pathway, often interacting with other proteins involved in the network. The importance of their role in the network led by p53 suggests that their dysregulation may have a major impact on tumorigenesis. As an example, the abnormal MDM2 expression associated with head and neck squamous cell carcinomas [114] comes along with compromised miR-143 and miR-145 activity [115]. Indeed, MDM2 is regulated by both miRNAs, whose maturation processes are in turn mediated by p53, thereby forming a FBL [116] that enhances proliferation suppression, cell cycle arrest, and apoptosis. When overexpression of MDM2 and poor expression of both miRNAs occur concomitantly, such functions are impaired and favor tumor initiation and development. Additionally, miR-1827 has been observed to repress MDM2 by binding to its 3’UTR [117], whereas MDM2, in turn, enhances the expression of p53. Interestingly, miR-1827 downregulation along with the consequent MDM2 overexpression has been linked to the growth of xenograft colorectal tumors. The proliferation of cancer cells has also been associated with two miRNAs that enhance cell quiescence in response to p53, favoring their re-entry into the cell cycle: miRNA-27b-3p and miRNA-455-3p [118].

In sum, the complex network that sees p53 as its core player is heavily interconnected with miRNA-mediated regulation. The alteration of p53 temporal trends, impairing its biological functions, is often underlined by miRNA dysregulation, which appears to be a direct source of tumorigenesis. Therefore, understanding the roles of miRNAs involved in the p53-mediated gene regulatory network and unveiling their targets might provide a useful contribution in the attempt to restore the tumor suppressor ability of p53.

### 3.2. microRNAs and Myc: Mediating Cell Proliferation and Differentiation

The MYC gene, also called c-Myc, encodes for the Myc TF that belongs to the family of helix-loop-helix leucine zippers [81]. Its transcription activation function is accomplished by the formation of heterodimeric complexes with Max proteins [119]. Myc is commonly known as a pivotal regulator of genes involved in cell fate decisions, such as cell proliferation, differentiation, and apoptosis [120,121,122,123,124], making it a key player in both the initiation and maintenance of tumorigenesis.

Genetic alterations of Myc gene such as mutations, chromosomal translocations, and gene amplification [125], have been observed to promote cell proliferation by allowing cancer cells to resume the cell cycle regardless of DNA damage [126,127] and accelerating cell division [128,129]. Coherently, Myc is known for repressing the expression of a core proliferation-inhibiting gene called CDKN1A [130]. Moreover, its overexpression increases telomerase activity, thus impairing the protective mechanism of telomere shortening and favoring cellular immortality [131].

Myc is also linked to cell fate decision mechanisms underlying cell differentiation: the activity of extracellular signal-regulated kinases (ERK), well-known for determining the choice between differentiation and proliferation, acts upstream of Myc. Indeed, Myc is directly targeted by ERK, while indirectly feeding back to it through the mediation of a pivotal cell cycle regulator, E2F1 [132,133].

The temporal pattern of ERK expression plays a crucial role in this discrimination. For instance, a nerve growth factor (NGF) signal induces a transient ERK response that leads to neuronal differentiation [134]. Conversely, an epidermal growth factor (EGF) signal causes pulsatile ERK expression that results in cell proliferation [135], whereas a physiological EGF level is associated to a more stochastic fashion of ERK activity, showing transient pulses [135,136]. As suggested by theoretical models, N-FBLs are at the origin of ERK oscillations [137].

**Figure 5:**
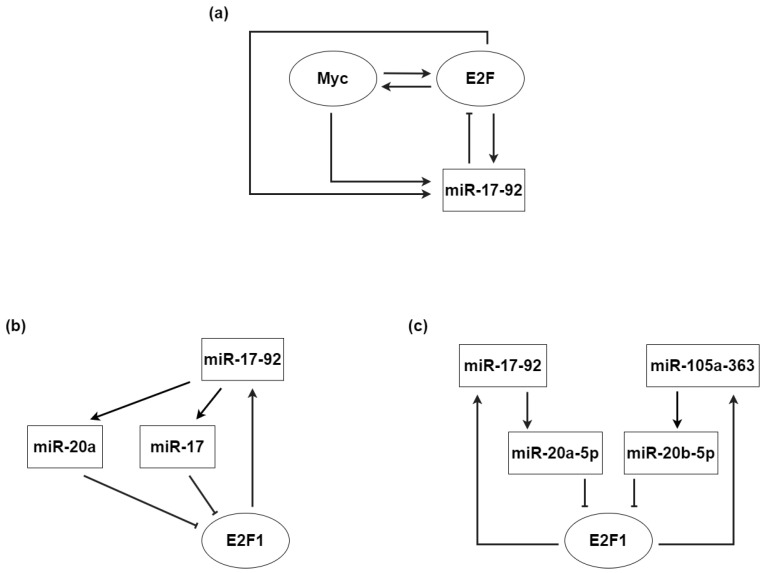
Representation of Myc-controlled networks involving miRNAs. Edges with a pointed head represent positive (activatory) regulation, whereas edges with a flat head represent negative (repressive) regulation. (**a**) Representation of regulatory interactions between Myc, the E2F family, and the miR-17-92 cluster. The miR-17-92 cluster can lead to the generation of large amplitude E2F1 oscillations thanks to the timescale gap between transcriptional and miRNA-mediated regulation. (**b**) Negative feedback loops between miR-17 and E2F1 and between miR-20a and E2F1 in mouse palatal mesenchymal cells. MiRNA-mediated repression avoids uncontrolled cell proliferation. (**c**) Negative feedback loops between miR-20a-5p and E2F1 and between miR-20b-5p and E2F1 in myoblasts. The two miRNAs promote cell differentiation.

Myc’s intricate interaction network involves the action of several miRNAs. Originally, it was shown that the miR-17-92 cluster is directly activated by Myc [138]. Later on, the miR-17-92 cluster was found to prolong cell cycle transitions between G1 and S phases [139]. Moreover, two miRNAs belonging to the same cluster, miR-17-5p and miR-20a, are now known to negatively regulate the expression of E2F1, whose transcription is enhanced by Myc [140] (Figure 5b). Thus, Myc directly activates E2F1 while indirectly inhibiting its translation through a miRNA-mediated I1-FFL.

In turn, as E2F1 feeds back to Myc, a P-FBL connects the two TFs. Moreover, E2F1 activates its own repressor cluster miR-17-92, thereby giving rise to an N-FBL [141]. The connections resulting from the combination of the I1-FFL with the two FBLs are depicted in Figure 5a. The inclusion of miRNAs in this network provides stability to both FBLs. In the case of the P-FBL between Myc and the E2F family, the miR-17-92 cluster contributes to a pulsatile TF expression that avoids uncontrolled proliferation by targeting E2F1. Indeed, the time delay introduced by miRNA-mediated regulation is able to induce large-amplitude oscillations in protein levels. Due to post-transcriptional repression, the rate of protein production does not depend on the mRNA concentration at the present time, but rather on its concentration at some past time point [142]. This mechanism allows large bursts of E2F1 protein expression, which can be exploited by the system to induce quick cell death responses. An increase in E2F1 and Myc levels is interpreted as an apoptotic signal, and thanks to a fast miRNA-mediated rise of protein levels, any danger caused by sudden and large perturbations is prevented.

On the other hand, the N-FBL between the miR-17-92 cluster and E2F1 is crucial for the discrimination between proliferation and differentiation in the regulatory network governed by Myc. In mouse palatal mesenchymal cells, miRNA-mediated E2F1 repression drives a controlled palatal development by preventing excess proliferation [143]. MiR-17 and miR-20a negatively target E2F1; after that, the latter induces the miR-17-92 cluster, thus regulating proliferation and cell cycle (Figure 5b). Instead, in myoblasts, the same miRNA cluster contributes, together with the miR-106a-363 cluster, to promote differentiation and repress proliferation [144]. Indeed, E2F1 activates the expression of both clusters, whose two members miR-20a-5p and miR-20b-5p concomitantly exert their repressive action on the TF (Figure 5c).

Interestingly, miRNAs modulate the epigenetic expression by targeting epigenetic-associated enzymes [145,146,147]. Moreover, miRNA expression is affected by regulation of epigenetic modifications, including DNA methylation [148], RNA modifications alarcon2015n, and histone modifications [149]. The mutual relation between epigenetic regulation and miRNAs results in the formation of a miRNA-epigenetic FBL . Such a miRNA-epigenetic FBL has been found to cooperate with Myc to control the stemness of mouse embryonic stem cells [150]. Sung-Hun Lee and colleagues uncovered an N-FBL where the protein arginine methyltransferase 7 (PRMT7) represses the miR-24-2 gene encoding miR-24-3p and miR-24-2-5p, and the latter targets, in turn, the 3’UTR of Prmt7. In addition, miR-24-3p and miR-24-2-5p target Myc. Therefore, PRMT7 antagonizes the downregulation of miR-24-3p and miR-24-2-5p against Myc, positively regulating its expression level in order to maintain mouse embryonic stem cells’ stemness.

In sum, miRNAs appear to play a crucial role in tuning the temporal expression features of several genes that represent the core of the Myc-controlled cell fate decision system, with remarkable connections to the E2F1 family of TFs. They are able to provide stability and robustness to Myc-mediated regulation, but their cooperation with its oncogenic potential can accelerate cancer development. This confers them a fundamental role in tumorigenesis, as shown in several studies [151,152]. For instance, miR-135b downregulation in osteosarcoma is associated with metastasis [153]. Data show that this miRNA acts as a tumor suppressor, regulating osteosarcoma proliferation, migration, and invasion by targeting Myc. Similarly, downregulation of the Myc-repressing miRNA miR-451 is associated with the migration and invasion of bladder cancer cells [154].

## 4. Conclusions

In this review, we addressed the tumorigenic role of miRNAs, resulting from their participation in the most significant cell fate decision mechanisms. In particular, we focused on their role in determining the dynamic features of temporal gene expression, both by analyzing theoretical regulatory motifs and reviewing their functions within endogenous p53- and Myc-governed networks. Transitions between cell states are determined by decoding the temporal dynamics of expression, and miRNAs act by providing stability and robustness to such time-dependent processes. By affecting gene expression, their dysregulation can either alter the topography of Waddington’s metaphoric epigenetic landscape or cause the cell to shift along the landscape toward abnormal cancer states, thereby favoring tumorigenesis.

The majority of studies here mentioned are limited to theoretical evidence. Indeed, the role of miRNAs within gene regulatory motifs characterized by time-dependent signals has been widely investigated in silico by mathematical modelling and computational simulation. The experimental validation of the resulting theoretical predictions is challenging, as remarkable precision and control are required, thereby making the development of sophisticated tools and techniques essential. In order to understand how miRNAs affect temporal gene expression, the ability to directly manipulate input signals to tightly control their temporal fashion is necessary along with a faithful monitoring of the responses of each motif element, including miRNAs, in time. For instance, spatially and temporally controlled dynamics can be investigated successfully by using optogenetic systems assisted by time-lapse fluorescence microscopy [155,156,157]. Optogenetic tools can be exploited to shape gene expression, allowing its spatial and temporal fine-tuning. Contrary to chemically controlled gene expression, optogenetics allows the reversible activation and inhibition of expression within a population of cells as well as single cells with a desired frequency and duration, thus providing a potential tool for temporally controlling miRNA-mediated motifs. Responses, intended as the expression levels of elements involved in the motif, can be monitored for instance by time-lapse fluorescence microscopy. MRNAs and protein products can be tagged with fluorescent proteins, and their dynamics can be recorded in real time by live-cell imaging, whereas miRNA dynamics can be followed by using systems such as MIRAR (miRNA activity reporter) [158]. In this system, a green fluorescent protein (GFP) reporter is fused to a 3’ UTR of human kras (Kirsten ras) gene. The kras gene is regulated by miRNA let-7; therefore, temporal GFPfluorescence intensity can be exploited as a proxy of let-7 activity. As Matthew A. Turk and colleagues demonstrated, this approach is generalizable to other miRNAs, and small changes in miRNA expression can be detected.

Once a strong experimental proof of miRNA mediation in shaping temporal gene expression is accomplished, the role of miRNAs within wider genetic networks can be assessed rigorously, thus providing a better understanding of how their dysregulation affects tumorigenesis and opening the way to new therapeutic strategies.
